# Impaired Cell Surface Expression of HLA-B Antigens on Mesenchymal Stem Cells and Muscle Cell Progenitors

**DOI:** 10.1371/journal.pone.0010900

**Published:** 2010-05-28

**Authors:** Adiba Isa, Jan O. Nehlin, Hardee J. Sabir, Tom E. Andersen, Michael Gaster, Moustapha Kassem, Torben Barington

**Affiliations:** 1 Center for Stem Cell Treatment, Department of Clinical Immunology, Odense University Hospital, University of Southern Denmark, Odense, Denmark; 2 Clinic for Molecular Endocrinology (KMEB), Department of Endocrinology, Odense University Hospital and Medical Biotechnology Center, University of Southern Denmark, Odense, Denmark; 3 Stem Cell Unit, Department of Anatomy, College of Medicine, King Saud University, Riyadh, Kingdom of Saudi Arabia; Centre de Recherche Public de la Santé (CRP-Santé), Luxembourg

## Abstract

HLA class-I expression is weak in embryonic stem cells but increases rapidly during lineage progression. It is unknown whether all three classical HLA class-I antigens follow the same developmental program. In the present study, we investigated allele-specific expression of HLA-A, -B, and -C at the mRNA and protein levels on human mesenchymal stem cells from bone marrow and adipose tissue as well as striated muscle satellite cells and lymphocytes. Using multicolour flow cytometry, we found high cell surface expression of HLA-A on all stem cells and PBMC examined. Surprisingly, HLA-B was either undetectable or very weakly expressed on all stem cells protecting them from complement-dependent cytotoxicity (CDC) using relevant human anti-B and anti-Cw sera. IFNγ stimulation for 48–72 h was required to induce full HLA–B protein expression. Quantitative real-time RT-PCR showed that IFNγ induced a 9–42 fold increase of all six HLA-A,-B,-C gene transcripts. Interestingly, prior to stimulation, gene transcripts for all but two alleles were present in similar amounts suggesting that post-transcriptional mechanisms regulate the constitutive expression of HLA-A,-B, and -C. Locus-restricted expression of HLA-A, -B and -C challenges our current understanding of the function of these molecules as regulators of CD8^+^ T-cell and NK-cell function and should lead to further inquiries into their expression on other cell types.

## Introduction

The highly polymorphic classical Major Histocompatibility Complex (MHC) class I antigens consist of extracellular membrane spanning heavy chains for HLA-A, -B, and -C, each in complex with a non-MHC subunit, beta-2-microglobulin (β2m). A major function of HLA molecules is presentation of intracellularily produced peptides to T-cell receptors (TcR) of cytotoxic CD8^+^ T lymphocytes which leads to killing of infected cells. In addition, HLA molecules act as ligands for the killer-cell immunoglobulin-like receptors (KIRs) on natural killer cells (NK) and NKT cells [1]. The Classical HLA class I can also act as ligands by having their leader peptides presented on non-classical HLA–E molecules to the CD94:NKG2A receptor on NK cells [2]. Furthermore, classical HLA class I molecules are ligands for certain members of the leukocyte immunoglobulin-like receptor (LILR/ILT/LIR) family, molecules with ill-defined regulatory functions in the immune system [3]. While it is well known that the three classical HLA class I loci and even alleles differ widely with respect to their interaction with KIR on NK cells [4], much less is known about differential effects of class I antigens on other aspects of the immune function.

Allele-specific down-regulation of HLA class I is well-known in cancer cells [5], [6], [7]. This is thought to result from genetic and epigenetic instability combined with selection due to immune recognition of tumour antigens and therefore may not be relevant for normal cells. Isolated expression of HLA–C by extravillous trophoblast is, however, well documented [8]. The fact that many viruses are able to establish chronic infections (e.g. HIV, HPV, and HBV [9], [10], [11]) and have developed mechanisms to down-regulate HLA class I expression in a locus-specific way could indicate differential functions of these molecules. Locus-specific expression is, however, rarely addressed in normal cells outside the haematopoietic system and almost all studies on HLA class I expression after birth have used a single antibody (W6/32) targeting simultaneously HLA–A, -B, -C and cross-reacting with HLA-E and –F [12]. These studies are therefore unable to address the differential expression of HLA class I loci [13]. Using such antibodies, human embryonic stem cells have been shown to express very low levels of HLA class I [14], [15], while lineage-committed stem cells like mesenchymal stem cells (MSC) have a much higher expression similar to that of lymphocytes [16]. However, later on during differentiation, HLA class I expression may be down-regulated or lost as suggested by the finding of failure to constitutively express HLA class I in several terminally differentiated cell types like neurons, hepatocytes, skeletal and cardiac muscle cells [17].

Human MSC represent a subset of stromal stem cells present in many adult tissues that have the potential to differentiate into various cell types including cells of the mesodermal lineage such as osteocytes, adipocytes and chondrocytes. They are identified by expression of different surface markers and by the lack of expression of the hematopoietic cell markers CD34, CD45 and CD14 [18], [19], [20]. MSC efficiently suppress alloimmune responses after transplantation due to their not fully understood immunomodulatory properties. They are known to express HLA class I antigens as judged by staining with the W6/32 HLA-ABC antibody, and expression of HLA class II molecules can be induced upon stimulation [21].

Human satellite cells are mitotically quiescent muscle progenitors present in adult muscles. When activated, they proliferate as muscle precursor cells which can fuse together and generate multi-nuclear myotubes which are immature myofibers or fuse to existing myofibers [22], [23], [24]. They have also shown the ability to differentiate to adipocytes and osteocytes [25].

In the present study we have investigated HLA-A, -B and -C expression using allele-specific antibodies and primer sets in MSC and human satellite cells.

## Results

### Mesenchymal stem cells and human satellite cells constitutively express high levels of HLA-A but not HLA-B measured by allele-specific flow cytometry

**Figure 1:**
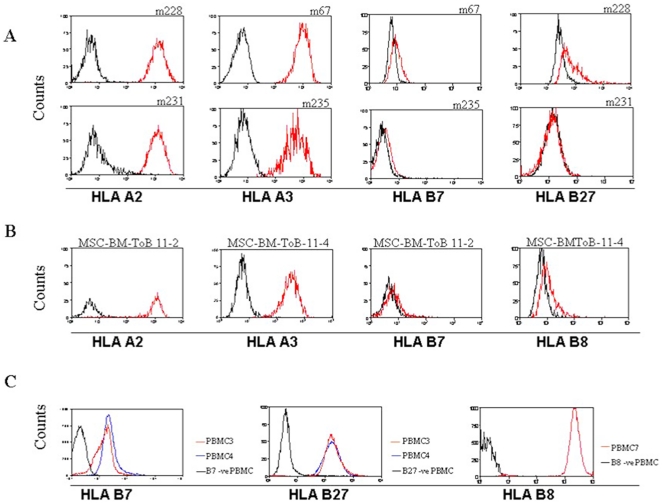
Comparison of constitutive allele-specific HLA class I expression on stem cells and PBMC. Flow cytometry analysis of allele-specific HLA class I expression. Histograms showing high expression of HLA-A2 and -A3 and a strongly downregulated HLA-B7, -B8 and -B27 expression on: (A) Four different primary human satellite cell lines, (B) Two bone marrow-derived primary MSC lines, (C) PBMCs from three different individuals.

All primary stem cell lines in this study were analyzed at passages 2–4 and showed high expression of HLA class I judged by direct staining with fluorochrome-conjugated anti-HLA-A,-B,-C antibody (W6/32). Allele-specific HLA-A2, -A3, -B7, -B8, and -B27 surface expression was measured on cells that were positive for these antigens by genomic tissue typing. HLA-A2 and -A3 were highly expressed (≥+2) on all cell lines and PBMCs carrying the corresponding genes, except one cell line that expressed somewhat lower levels of HLA-A2 (+1) (Table 1 and Figure 1). Surprisingly, cell-surface expression of all three analyzed HLA-B proteins were either very low or absent on all 13 analyzed stem cell lines (Figure 1A and B). We could not investigate the occurrence of allele-specific HLA-Cw surface expression by flow cytometry due to the lack of available allele-specific HLA-Cw antibodies. HLA-DR was also analyzed by flow cytometry and in accordance to previous data [16] it was not expressed on stem cells, although, it could be induced on both MSC and human satellite cells by IFNγ stimulation (data not shown).

**Table 1:** Cells and cell lines of this study, their genomic types and semi-quantitative cell-surface expression of HLA class I.

Cell ID.	Source	Cell Type	Genomic HLA Type			Cell-surface expression^a^				
			A	B	C	A2	A3	B7	B8	B27
MSC-Tert clone DD8	Bone marrow	Mesenchymal stem cell Tert transfected	02, 03	07, 27	07, 02	3+	2+	+/−		+/−
MSC-DF	Bone marrow	Mesenchymal stem cell	02, 02	07, 44	05, 07	4+		+/−		
MSC TOB 11-02	Bone marrow	Mesenchymal stem cell	02, 03	07, 40	03, 07	4+	NA	-		
MSC TOB 11-04	Bone marrow	Mesenchymal stem cell	01, 03	08, 57	06, 07		3+		-	
MSC-AT-661	Adipose tissue	Mesenchymal stem cell	01, 23	08, 14	07, 08		4+		+/−	
m54	Muscle	Human satellite cell	03, 25	18, 27	01, 12		NA			1+
m67	Muscle	Human satellite cell	03, 29	07, 44	07, 16	3+		-		
m226B	Muscle	Human satellite cell	02, 03	07, 51	07, 14	1+	NA	-		
m228	Muscle	Human satellite cell	02, 02	27, 40	02, 03	3+				1+
m231	Muscle	Human satellite cell	02, 02	15, 27	02, 03	2+		-		1+
m233	Muscle	Human satellite cell	03, 03	07, 14	05, 07		2+	-		
m234	Muscle	Human satellite cell	03, 29	07, 44	07, 16		3+	-		
m235	Muscle	Human satellite cell	03, 68	07, 39	05, 07		2+	1+		
PBMC 3	Peripheral blood	Lymphocyte	02, 03	07, 27	07, 02	4+		1+		2+
PBMC 4	Peripheral blood	Lymphocyte	03, 26	07, 27	07, 02		NA	2+		2+
PBMC 7	Peripheral blood	Lymphocyte	02, 26	08, 27	01, 07	4+			4+	2+

^a^Surface expression based on direct flow cytometry, only data from relevant combinations of antisera and genomic types are shown. NA =  Not Analyzed, “−” =  the specific mean fluorescence intensity (MFI) is ≤3 times higher than the MFI of the isotype control “+/−” = <10 times, “1+” =  ≥10 times, “2+” =  ≥30 times, “3+” =  ≥50 times and “4+” =  ≥100 times.

### Induction and kinetics of HLA-B surface expression on human stem cells by IFNγ stimulation

**Figure 2:**
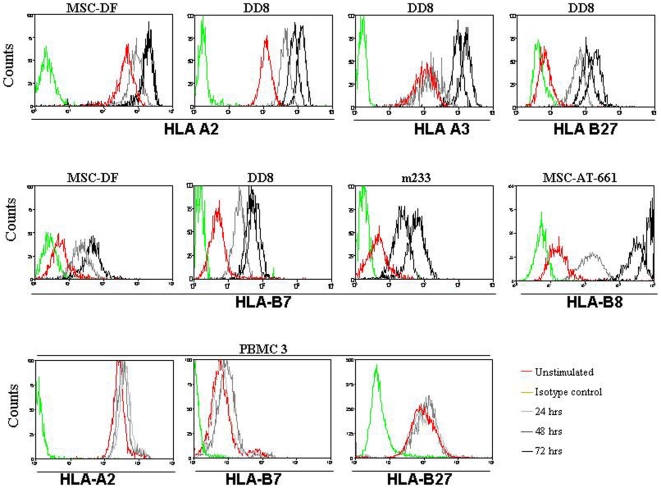
Kinetics of HLA-A and -B upon IFNγ stimulation of MSC and human satellite cells. Overlay histograms showing expression of HLA class I alleles before and after IFNγ (25 ng/mL) stimulation. Kinetics (0 h, 24 h, 48 h and 72 h) is shown for different alleles, different MSC lines, human satellite cells and PBMC.

We further explored whether the low-expressed HLA-B alleles could be up-regulated on the cell-surface by stimulation with rhIFNγ. One cell line from each cell type, DD8, MSC-DF, MSC-AT-661 and human satellite cell m233 and PBMC from donors 3 (Table 1 and Figure 2) and 4 (data not shown) were stimulated for 24, 48, and 72 h, respectively, with rhIFNγ (25 ng/mL) and analyzed by flow cytometry with fluorochrome-conjugated allele-specific antibodies. The constitutive expression of HLA-A2 and -A3 was already high on DD8 cells, however, after 72 h of IFNγ stimulation, a further ∼10-fold increase was seen (Figure 2). HLA-B7 was very low and HLA-B27 was virtually absent on DD8 cells, but IFNγ stimulation induced both alleles' expression already after 24 h, and this up-regulation continued to 72 h. Additional four days of stimulation did not increase the expression further (data not shown).

Similar results were found for bone marrow-derived MSC-DF cells (HLA-B7), adipose tissue-derived MSC-AT-661 (HLA-B8), and human satellite cells m233 (HLA-B7) (Figure 2). In contrast to the up-regulation of HLA class I on stem cells, HLA-A and -B alleles were already highly expressed on PBMC3 and PBMC4 and no further up-regulation of HLA-A2, -B7 or -B27 could be measured after 48 h of IFNγ stimulation (Figure 2).

### Down-regulation of HLA-B expression is re-established after abrogation of IFNγ stimulation

In order to see if IFNγ-induced HLA-B expression was reversible, we measured the kinetics of cell-surface HLA-A2, -A3, -B7 and -B27 expression up to 96 h after removal of IFNγ. DD8 cells were stimulated with IFNγ for 72 h, hereafter IFNγ was washed away and the kinetics of cell-surface expression of HLA-A and -B was measured daily by flow cytometry. Already after 24 h, down-regulation of HLA-A and -B was noticed. The cell-surface expression of HLA-A and -B was decreased to the levels of the non-induced conditions 72 h after removal of IFNγ (data not shown).

### Determination of cell-surface HLA protein expression by quantitative flow cytometry

**Figure 3:**
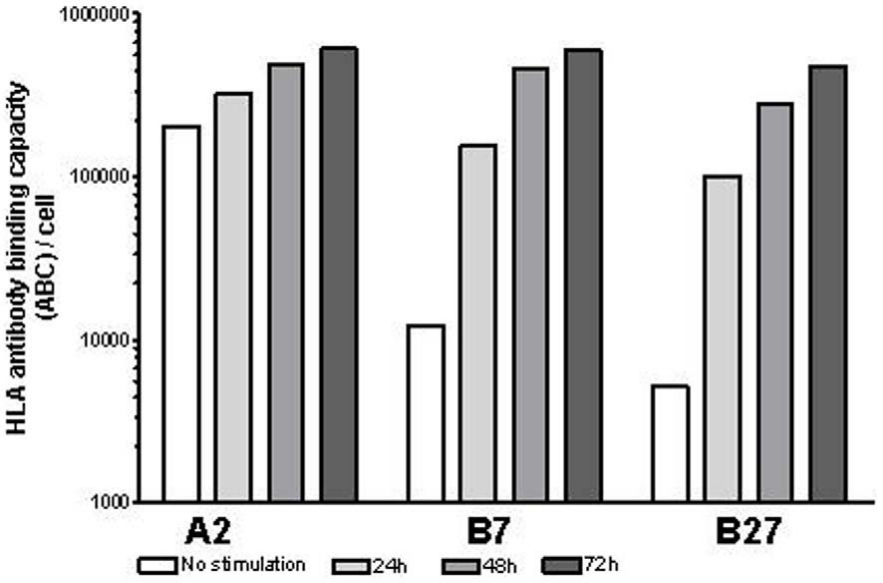
Kinetics of allele-specific HLA class I surface expression. Quantitative measurements of constitutive and up-regulated allele-specific HLA class I protein expression pre- and post-IFNγ stimulation of DD8 cells measured by indirect flow cytometry.

For an accurate comparison and quantification of simultaneous cell-surface expression of different HLA alleles on the same cell line, we further measured allele-specific HLA-A2, -B7 and -B27 expression by indirect immunofluorescence staining and using a standard curve based on beads conjugated with different known numbers of murine IgG molecules and stained with the same secondary antibody (see M&M). In un-stimulated cells, HLA-B7 and -B27 proteins were 17- and 40-fold less expressed, respectively, compared to the expression of HLA-A2 protein and HLA-B27 was 2.3 times less expressed than -B7. In comparison, IFNγ-stimulation increased HLA-A2 expression only 3-fold (Figure 3 and Table 2) and at the peak of IFNγ stimulation (∼72 h), HLA-B7 and -B27 surface expression had increased about 50 and 92 times, respectively (Figure 3).

**Table 2:** Primer sequences and quantitative allele-specific HLA-class I mRNA expression data on DD8 cells.

Primer^a^	Primer sequence (5′-3′)	Fragment length (bp)	mRNA copy no.^b^	Constitutive surface expression ABC^c^
FBXL12-143F d	ACCTGACGCTCTACACGATGC	108	NA	NA
FBXL12-251R	GAGCCAGAGAACAGGTAGCCA			
HLA A-minus2F	GGATGGCCGTCATGGCGCC	1,065	NA	NA
HLA A-1063 R	CCTGGGCACTGTCACTGCTT			
HLA B-minus40F-5 UTR	CACGCACCCACCCGGACTCA	1143		NA
HLA-B-1091R-3 UTR	TTTCAAGCTGTGAGAGACACATCA			
HLA-Cw-minus49F-5UTR	TAAAGTCCCCAGTCACCCACC	1,122	NA	NA
HLA Cw-1073 R	TCAGAGCCCTGGGCACTGTT			
HLA-A020101-272F	TGAAGGCCCACTCACAGACTC	111	8.9	204,370
HLA-A020101-383R	CCCACGTCGCAGCCATACATT			
HLA-A0301-508F	AAGTGGGAGGCGGCCCATGA	128	10.7	NA
HLA-A0301-636R	ATGTGTCTTGGGGGGGTCCGT			
HLA-B0702-522F	CCGTGAGGCGGAGCAGCG	99	30.1	12,076
HLA-B0702-621R	GTCAGCGCGCTCCAGCTTG			
HLA-B2702-232F	GGGACCGGGAGACACAGATC	105	41.8	5,174
HLA-B2702-337R	CGCTCTGGTTGTAGTAGCGGA			
HLA-Cw020202-101F	CCGCTGTGTCCCGGCCCA	117	18.6	NA
HLA-Cw020202-218F	GCCCGCGGCTCCCCTCTT			
HLA-Cw0702-972F	TGGAGCTGTGGTCACCGCTA	95	5.4	NA

^a^The numbers refer to the start of the primer relative to +1 ATG.

^b^mRNA Copy no./Reference gene mRNA copy no. measured in the same sample for each allele, see Materials and Methods.

^c^Antibody Binding Capacity (QifiKit), antibodies bound per cell from indirect flow cytometry.

^d^FBXL12 is the reference gene.

NA =  Not Analyzed.

### HLA-B molecules are not retained intracellularily

In order to investigate whether HLA-B protein is synthesized within the cells but is prevented from reaching the cell surface in the absence of IFNγ, we performed intracellular staining to detect HLA-A and -B proteins within DD8 cells. As a positive control for intracellular staining, the cells were incubated with un-conjugated anti-prolyl-4-hydroxylase antibodies, a protein normally present in the endoplasmic reticulum, followed by incubation with a secondary fluorochrome-conjugated antibody. We did not detect any intracellular HLA-B7 and -B27 proteins, however, a weak HLA-A2 protein expression could be detected (data not shown).

### Low constitutive expression of HLA-B and -C hamper complement-dependent cytotoxicity

**Figure 4:**
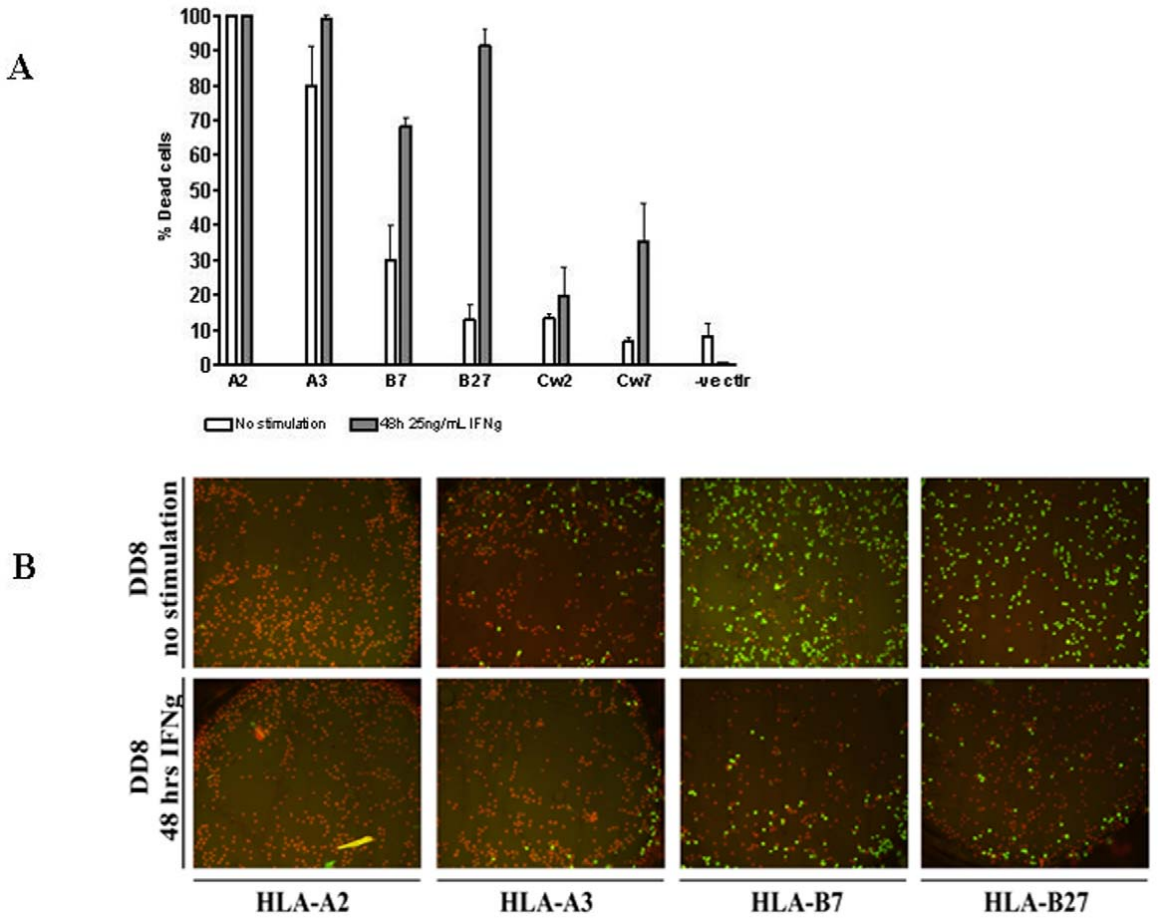
Analysis of allele-specific HLA class I expression performed by complement-dependent microcytotoxicity assay. (A) Histogram showing percentages of dead cells from three independent experiments. (B) Images showing CDC results for HLA-A2, -A3 and -B7 and -B27 for IFNγ-stimulated and non-stimulated DD8 cells. Green: living cells, red: dead cells.

Using a routine serological complement-dependent cytotoxicity (CDC) typing method [26] with allele-specific immune sera and rabbit serum as the source of complement, we were able to confirm the expression of HLA-A on all stem cells tested by their massive killing (96%) with relevant anti-HLA-A anti-sera but not by human AB serum (average 9%). In contrast, impaired killing was observed for all HLA-B alleles tested (-B7, -B8, -B14 and -B27); only about 28% of the MSC were recognized and killed with anti-HLA-B anti-sera, which is not sufficient for typing as positive (>50% killing) by standard criteria for this assay. Furthermore the recognition and killing of MSC by anti-sera containing anti-HLA-Cw (Cw2 and Cw7) antibodies were even lower (9%) i.e. the cells were typed negative for HLA-Cw (Figure 4A).

IFNγ-stimulation for 48 h was sufficient to allow positive test results for HLA-B and -Cw. Under these circumstances, the killing of MSC by anti-HLA-B and anti-HLA-Cw antibodies was 81% and 26%, respectively (Figures 4).

### Determination of quantitative allele-specific HLA-A, -B and -C gene transcripts

High-resolution typing of HLA-A,-B,-C in DD8 cells was performed and confirmed by cloning and sequencing the relevant exons within the HLA loci. Based on the high resolution type, we optimized allele-specific real time-PCR for measuring mRNA expression of six HLA alleles: A*02:01:01, A*03:01:01, B*07:02:01 and B*27:02, C*02:02:02 and C*07:02 (Materials and methods). We tested seven potential reference genes, and chose *FBXL12* since it was only minimally influenced by IFNγ stimulation (data not shown). All real time-PCR-amplified cDNA fragments of MSC line DD8 cell cultures were tested for purity by gel electrophoresis, cloned and sequenced to confirm allele specificity.

**Figure 5:**
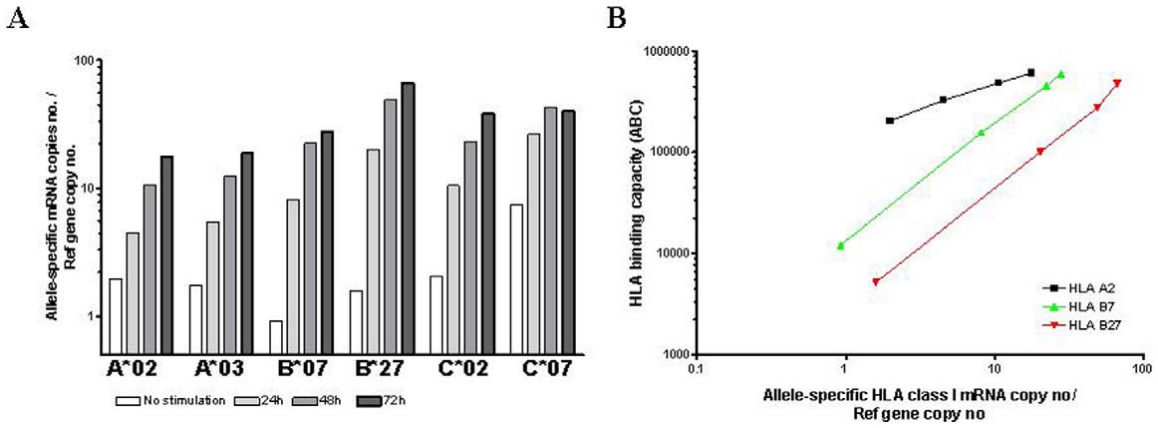
Kinetics of HLA class I gene transcripts upon IFNγ stimulation and correlation between allele-specific surface protein expression and mRNA expression. (A) Quantitative measurements of constitutive and up-regulated allele-specific HLA class I gene transcripts pre- and post-IFNγ stimulation by quantitative RT-PCR for DD8 cells. (B) Correlation between constitutive and up-regulated expression of HLA-A and -B allelic gene transcripts and the capacity of antibody binding to the surface HLA class I allelic proteins by indirect flow cytometry staining for HLA-A2, -B7 and -B27. HLA-B*07 showed the lowest constitutive mRNA expression while HLA-B27 showed the lowest constitutive protein expression.

In order to understand the mechanism(s) behind the low constitutive HLA-B cell surface expression, we proceeded to measure allele-specific gene transcripts in DD8 cells. Allele-specific mRNA was detected for all six alleles and expressed as a geometric mean of copy number of the target HLA allele/copy number of the reference gene from three independent experiments. Prior to IFNγ stimulation and in striking contrast to the vast differences in constitutive cell surface expression documented by flow cytometry (Figure 2 and Table 2), transcripts of HLA-A*02, -A*03, -B*27 and -C*02 were expressed at similar levels (ratios: 1.6–2.0) (Figure 5A). Moreover, expression of the B*07 gene transcript was only slightly lower than HLA-A*02 transcripts (0.9, p<0.01, Student t test). In contrast, the constitutive (un-induced) expression of HLA–C*07 was significantly higher than all other five HLA class I alleles (7.4, p<0.01).

Stimulation with IFNγ increased mRNA levels dramatically already after 24 h culminating at 72 h (Figure 5A). HLA–B*27 increased 42-fold and ended up with the highest mRNA level of all HLA class I alleles (∼4-fold more transcripts than HLA-A*02) while HLA-B*07 increased 30-fold and HLA-A*02 only about 9-fold (Figure 5A and B). The increases in mRNA levels correlated well with increases in surface expression of the corresponding HLA alleles (A2, B7, B27) (Figure 5B).

The expression of HLA class I allele-specific mRNA was further tested up to 96 h after removal of IFNγ. In parallel with cell surface expression, HLA class I allele-specific mRNA expression levels decreased dramatically 48 h after IFNγ removal and reached at 72 h almost to the same levels as in un-stimulated conditions (data not shown). Though this in itself strongly suggested that copy numbers detected by qRT-PCR reflected full length transcripts with the potential for translation, we wanted to exclude that a predominant splice variant might interfere with these results. We therefore performed long-range locus-specific RT-PCRs comprising all exon-exon junctions for all three HLA loci. Agarose gel electrophoresis revealed bands of the expected sizes and these fragments were purified, cloned, sequenced and found to contain all expected exons (1–8 for HLA-A and -C, 1-7 for HLA-B, data not shown). The results showed no indication of the presence of a quantitatively significant splice variant within any of the three HLA loci, neither in un-induced nor in stimulated conditions. Some minor bands were seen though. A few of these were purified and sequenced directly. Only PCR artefacts due to mispriming were found.

## Discussion

Human embryonic stem cells and trophoblasts have been shown to express very low levels of HLA class I [14], [15]. However, more differentiated stem cells such as human mesenchymal stem cells and human satellite cells express significant levels of HLA class I, and have the ability to express HLA class II upon IFNγ stimulation [16], [27]. Interestingly, MSC have been reported to down-regulate HLA class I expression after differentiation to the three different lineages, adipocytes, chondrocytes and osteocytes and loose the ability to up-regulate their HLA class I upon IFNγ stimulation in the process [16]. This indicates a hitherto largely unrecognized complexity in developmental regulation of HLA expression and it is unknown if the three classical HLA class I molecules follow the same developmental pattern or deviate from each other.

In the present study we have investigated the constitutive allele-specific expression of classical HLA class I on human MSC and human satellite cells, both at the protein and mRNA levels. Both cell types were strongly positive for the commonly used monoclonal HLA-A,B,C antibody (clone W6/32). However, using allele-specific antibodies targeting classical HLA class I molecules, we found that there is a selective cell-surface expression of HLA-A on these cells while HLA-B was highly down-regulated (Figure 1). To the best of our knowledge, this is the first time a differential locus-specific HLA class I expression is reported on normal human cells and this contrasts to the approximately equal expression of HLA-A, -B, and -C found on lymphocytes.

Furthermore, we investigated whether stem cells had the capacity to up-regulate the surface expression of HLA-A and -B alleles upon IFNγ stimulation. Indeed, all stem cells up-regulated their expression of HLA-A and -B with the highest fold increase measured by quantitative flow cytometry during the first 24 h of stimulation and peaking after 72 h. In contrast, PBMC did not show any further up-regulation of HLA-A or -B (Figure 2). The failure to express HLA-B on the surface of MSC was not due to intracellular retention of protein, because no intracellular expression of HLA-B protein could be detected in DD8 cells during un-induced conditions. However, a weak intracellular HLA-A2 expression was monitored in un-stimulated DD8 cells (data not shown).

The constitutive expression of HLA-A and down-regulation of HLA-B proteins were confirmed by complement-dependent cytotoxicity tests (CDC). In accordance with flow cytometry data, stem cells expressing high levels of HLA-A proteins were killed when using human antisera containing HLA-A2 and -A3 antibodies (100% and 80% lysis, respectively). In contrast, stem cells were relatively resistant to killing when using antisera containing HLA-B and -C allele-specific antibodies, and only 4–40% of the cells were killed (Figure 4A). This means that MSC actually would have typed negative for the HLA-B types by standard criteria for serological tissue typing. However, after 48 h of stimulation with IFNγ, MSC were shown to be more susceptible to lysis compared to non-stimulated cells confirming the up-regulation of HLA-B and suggesting a concurrent up-regulation of HLA-C on these cells (Figure 4B).

We investigated whether the strong down-regulation of HLA-B surface expression was due to the lack of HLA-B gene transcription. Surprisingly, quantitative allele-specific HLA class I RT-PCR data showed that gene transcripts were expressed at the same levels in four out of six alleles (HLA-A*02, -A*03, -B*27 and -C*02) in basal conditions, thus yielding no explanation for the vast difference in surface expression. In comparison, HLA-B*07 and HLA–C*07 gene transcripts were expressed significantly lower and higher, respectively (p<0.001, two-sample t test), but even for HLA-B*07 the relative mRNA level (2-fold lower than that of HLA-A*02) could not explain the low surface expression of HLA-B7. Thus, we considered the possibility that this could be an artefact due to the presence of different splice variants being detected by RT-PCR but not able to generate functional proteins as it has been shown previously in some cancer cell lines [28], [29]. However, PCR amplification of full-length mRNA products ruled out this possibility, since no significant splice variants of HLA class I gene with potential of being amplified in our qRT-PCR were detected.

HLA-A and -B have been reported to be regulated primarily at the transcriptional level through promoter elements that are conserved among the genes [30], [31]. The induction of gene transcription by IFNγ has been shown to be mediated by the IFN-stimulated response element (ISRE) which is located in the 5′ promoter. Transcriptional control has also been reported in trophoblasts for HLA-A and –B [8], [16], [31], [32]. Recent data, however, suggest that post-transcriptional regulation can also be involved in the response of HLA-A to IFNγ stimulation [33]. At the mRNA level, all tested alleles in the present study showed inducibility by IFNγ, albeit at different levels, and the HLA-B alleles studied were highly responsive to IFNγ stimulation. HLA-B*27 mRNA demonstrated the highest fold increase (x42) after 72 h of IFNγ stimulation ending 4-fold higher than HLA-A*02 or -A*03 mRNA's (Figure 5A and Table 2). Previous studies have shown that the HLA-B locus contains a much stronger ISRE than the HLA-A locus which could be one of the reasons why HLA-B genes are more inducible than HLA-A [34], [35]. Accordingly, other studies have shown that HLA-A genes are either very low or not inducible by IFNγ stimulation [34]. However we could measure a 3-fold up-regulation of HLA-A genes upon 72 h of IFNγ stimulation on DD8 cells. A proportional correlation could also be measured between HLA class I allele-specific gene transcript and cell-surface HLA class I antigen expression upon IFNγ stimulation (Figure 3) which confirms the presence of transcriptional regulation during IFNγ stimulation in accordance with previously published data [36].

Competitive mRNA PCR data have previously been presented regarding the differential mRNA locus-specific expression of HLA class I mainly in cancer cell lines [30]. However, in the present study we measured allele-specific HLA class I gene transcript expression in DD8 cells. We have detected almost equal levels of mRNA expression for all six but two alleles, despite the impaired cell-surface expression of HLA-B and possibly HLA-C (based on CDC data) antigens. Our current data here suggest the possible involvement of post-transcriptional regulation inhibiting translation of HLA-B and possibly HLA-C surface antigens in the absence of IFNγ stimulation. The great discrepancy between the constitutive locus-specific surface expression found amongst HLA-A,-B and -C could possibly be explained by translation attenuation by microRNA(s) specific for HLA-B (and possibly -C) alleles as it has been shown for HLA-G expression [37].

Lack of constitutive HLA-B expression in normal cells challenges current hypotheses in immunology. E.g. according to the missing self hypothesis of NK-cell activation absence of HLA-B expression should turn stem cells into targets of NK cell-mediated killing in most HLA-Bw4 positive individuals who usually carry this allotope on HLA-B (e.g. HLA-B27) and who usually carry significant numbers of NK cells solely expressing the killer inhibitory immunoglobulin-like receptor (KIR) 3DL1 which is dependent on recognition of Bw4 for inhibition of killing [38], [39]. If the down-regulation of HLA-B is a more general phenomenon and not restricted to stem cells it might even be related to the apparently different roles of classical HLA antigens in antimicrobial immunity that are currently emerging. For instance, immune responses to HIV, EBV and *Mycobacterium tuberculosis* are often dominated by T cells specific for a few epitopes which tend to be presented by HLA-B rather than by HLA-A or –C [10], [40], [41]. HLA genotypes have also turned out to be important in influencing outcomes of infections such as HIV and HCV [41]. Moreover, a clear association between HLA-B*27 genotype and clearance of hepatitis C virus infection has been reported [42]. Increasing evidence indicates that there are two different subsets within the CD4^+^ and the CD8^+^ T cells: effector T cells and polyfunctional T cells. Polyfunctional CD8^+^ T cells are more capable of producing IL-2 and IFNγ, and are associated with stronger immune responses with efficient antiviral immunity than effector T cells. Interestingly, studies have shown a tendency for polyfunctional T cells to be specific for HLA-B restricted peptides [43], [44], [45]. Furthermore, the tolerance mediating protein, programmed death-1 molecules (PD-1) is significantly up-regulated in HLA-A restricted effector cells compared with HLA-B restricted polyfunctional CD8+ T cells with low level expression of PD-1 [43].

An intriguing possibility is that lack of constitutive HLA-B expression could be involved in the above mentioned mechanisms by influencing the induction of peripheral tolerance towards HLA-B restricted peptides.

In conclusion, our data here uniformly show that HLA-A is highly expressed on human mesenchymal stem cells and human satellite cells. Simultaneous expression of HLA-B is either very low or absent on these cells. Further investigations will help to elucidate the mechanism(s) that influence the differential expression of HLA-A and -B (and possibly -C) in stem cells and in differentiated cells and their possible role in the regulation of CD8^+^ T-cells.

## Materials and Methods

### Cell lines and cell culture

Donors and cell lines were selected based on genomic HLA-A and HLA-B types to assure that allele-specific antibodies were available for at least one A and one B allele of each cell (Table 1).

### Human mesenchymal stem cells derived from bone marrow and adipose tissue

Primary human MSC derived from bone marrow (MSC-BM) were established from bone marrow aspirates from four donors as described previously [16]. Briefly, primary hMSC were cultured in minimum essential medium (MEM) with low glucose (1 g/L) containing 2 mM L-glutamine (Invitrogen), supplemented by 10% fetal bovine serum (FBS, Invitrogen), 100 U/mL penicillin and 100 µg/mL streptomycin (Invitrogen). Adherent colonies were detached by treatment with D-PBS based TryPLE Express (Invitrogen) containing a trypsin-like enzyme and 1 mM EDTA for 5 min at 37°C, and subsequently re-plated for continued passage 1:2. Medium was changed twice weekly and cultures were maintained at 37°C in a humidified atmosphere at 5% CO_2_. A telomerase-immortalized subclone hMSC-Tert4 clone DD8 (DD8) cells [46] were used while the remaining cell lines were used as primary cultures at passages 2–4. Two MSC cell lines derived from adipose tissue (MSC-AT) were obtained from subcutaneous fat from two individuals undergoing liposuction at a plastic surgical clinic, Odense, Denmark and established according to the previously published method [47]. Briefly, the tissues containing MSC-AT were washed several times in PBS [pH 7.4] Ca++, Mg++ (Invitrogen) to remove red blood cells and digested with 200 U/mL collagenase I (Sigma) in 3 mM CaCl_2_ (Sigma) for 60 min at 37°C. Subsequently, the suspensions were centrifuged and pellets were washed once in αMEM (Invitrogen). Prior to culture, the cells were filtered through a 300 µm nylon mesh to remove fibrous tissue and cells were seeded at passage 0 (5–7×10^3^ cells/cm^2^). MSC properties were confirmed by flow cytometry showing high expression of CD44, CD73, CD105, CD166 and CD146, and no expression of CD34, and by the capacity to differentiate into the three lineages *in vitro*: osteoblasts, adipocytes, and chondrocytes [25].

### Human satellite cell derived from muscle biopsies

Human satellite cell cultures were established from quadriceps femoris muscle biopsies as previously described [48], [49]. The stem cells used in this study were stimulated by recombinant human interferon gamma (rhIFNγ) (Invitrogen) at a final concentration of 25 ng/mL since higher concentrations of rhIFNγ did not result in higher HLA up-regulation (data not shown).

### Flow cytometry

Cells were trypsinized and washed twice with PBE (PBS containing 2 mM EDTA (Ambion) and 5% fetal bovine serum (FBS, Invitrogen)). The cells were stained with the appropriate monoclonal antibodies (mAb's) (see detailed information in supporting Table S1), incubated for 20 min in dark on ice. The cells were washed twice with PBE and fixed in 1% formaldehyde in PBS (Bie Berntsen). Intracellular staining was performed with BD fixation and cytoperm kit (BD Biosciences), according to the manufacturer's instructions. When measuring the presence of intracellular HLA-A2, -B7 and -B27 proteins, the extracellular HLA-A2 proteins were blocked with purified un-conjugated anti-HLA-A2 antibodies, followed by staining with fluorochrome-conjugated antibodies against HLA-A2, HLA-B7 and HLA-B27 for 20 min, in the dark on ice followed by 2 washes (with 1x BD Perm/Wash™ buffer) and fixation (Table S1). Quantification of surface bound allele specific HLA molecules on each cell was performed by QifiKit (Dako) using indirect immunofluorescence staining. Primary un-conjugated antibodies against HLA-A2 (0.05 mg/mL), HLA-B7 (0.1 mg/mL) and HLA-B27 (10 µL) were carefully titrated. Cells were incubated with the antibodies 30 min on ice followed by 2 washes with PBE. Thereafter, cells were stained with the same FITC-conjugated polyclonal rabbit anti-mouse antibodies as above. The quantity of the bound primary antibodies was estimated using a standard curve for beads with different known numbers of murine IgG molecules stained with the same secondary antibody.

PBMC from three HLA class I type-selected donors were used as positive controls (Table 1). Each staining was performed in parallel with Ig isotype controls, which in all cases were shown to be negative except in the FITC channel in which all stem cells showed low-intensity autofluorescence. Blockade of Fc receptors by incubation of the cells with mouse serum did not affect the signals of the isotype controls (data not shown). Cell acquisition was performed with CyAn-ADP (Beckman Coulter) using Summit 4.3.1 software (Dako). Immediately, prior to cell acquisition, 150 µL of TryPLE express (Invitrogen) was added to the cell suspension to avoid clotting in the CyAn. The flow cytometer was standardized by using positive and negative controls and calibration beads each time to reduce day-to-day variation.

### Complement-dependent microcytotoxicity assay (CDC)

HLA-A, -B and -C surface expression and the lack of expression were confirmed by using the traditional CDC assay, using allo-antisera in Terasaki trays, as previously described with some minor modifications [26]. Briefly, the cells were re-suspended in 100 µL of Terasaki and Park medium (including 10% heat inactivated human AB serum). One µL of the cells was plated and incubated for 30 min at room temperature (rt) with equal amounts of allo-antisera. Subsequently 2 µL of rabbit complement mixture (1 mL freshly used complement, supplemented with 15 µL mixture of 0.03% acridine orange and 0.1% ethidium bromide (Ampliqon)) was added to the cells, mixed and incubated at rt in the dark for 60 min. As positive control we stained the cells either with a pool of HLA-A antibodies or PBMCs expressing the corresponding HLA alleles. As negative, we incubate the cells with human AB+ serum in each staining. The cytotoxicity rate was determined by quantitative analysis using an inverted fluorescence microscope DM-IRB with a DAPI filter (Leica). All samples were run in duplicates in 3 experiments and were scored by randomized and blinded counting; at least 200 cells per well were counted. Cells positive for a specific allele stained red, since the cells were lysed and EtBr was incorporated in the nucleus. In contrast, cells which did not express the corresponding HLA allele had an intact cell membrane and stained green under the microscope.

### HLA typing

Genomic DNA was purified from cells with the QiaAmp DNA Mini-kit (Qiagen). Low-resolution HLA class I types were determined using the LabType SSO (Sequence-specific oligonucleotide probes) typing test (One Lambda) using a Luminex 100 IS (Luminex Corp.) flow analyzer as recommended by the manufacturers (Table 1).

### RNA isolation and cDNA synthesis

Total RNA was isolated from DD8 cells using the RNeasy Plus mini RNA purification kit (Qiagen) and quantified by NanoDrop spectrophotometry (Thermo Fisher Scientific). The cDNA was synthesized using the RevertAid H Minus first strand cDNA synthesis kit with random hexamer primers (Fermentas).

### High resolution typing and full-length cloning of allele-specific HLA class I genes and transcripts

PCR primer pairs were designed to amplify HLA-A, -B, -C locus-specific fragments and -A*02, -A*03, -B*07, -B*27, -C*02, -C*07 allele-containing cDNA, synthesized from 50-100 ng of total RNA. PCR was performed either using AmpliTaq Gold DNA polymerase (Applied Biosystems) or Herculase II Fusion DNA polymerase with dNTPs combo kit (Stratagene). The reactions were incubated in a Mastercycler (Eppendorf) 5–12 min at 95°C, followed by 40 cycles (30–60 sec at 95°C, 30–60 sec at 66°C and 1.5–2 min at 72°C) and one final 10 min step at 72°C. The DNA was purified using the GFX PCR DNA and gel band purification kit (GE Healthcare) and sequenced using the same primers as used during PCR-amplification, using an ABI PRISM 3130*xl* genetic analyzer (Applied Biosystems) according to the published method [50]. When direct fragment sequencing was not possible, cDNA fragments encoding HLA alleles were ligated into a pJET1.2/blunt cloning vector using the CloneJet PCR cloning kit (Fermentas) and transformed into *E. coli* strain TOP10 (Stratagene). Single colonies were grown to isolate plasmid DNA using a plasmid purification Mini-kit (Qiagen) followed by sequencing.

The full-length sequences of DD8 HLA alleles can be retrieved from the IMGT/HLA database at: http://www.ebi.ac.uk/imgt/hla/allele.html [51].

### Allele-specific HLA class I quantitative RT-PCR

Primers for real-time PCR were designed to amplify fragments corresponding to the six HLA-A, -B, -C alleles of DD8 cells. Only primers which generated a single fragment of the predicted size judged by agarose gel electrophoresis and melting curve analyses were considered. Furthermore, allele specificities were confirmed by direct sequencing of the products. Seven different reference genes were tested; amongst them *FBXL12* was chosen since its expression showed similar C_T_ values to un-stimulated HLA-A transcripts and low responsiveness to rhIFNγ [52]. The PCR reaction contained SyBr green PCR master mix (Applied Biosystems), 0.25 µM of each primer (see Table 2) and cDNA (corresponding to 25 ng of total RNA). Quantitative PCR was performed in a 7300 Real-Time PCR system (Applied Biosystems). The reactions were first incubated 2 min at 50°C, and 10 min at 95°C, followed by 40 cycles (15 sec at 95°C, 30 sec at 66°C and 1 min at 72°C) and 10 min at 72°C, and a final DNA dissociation step. C_T_-value standard curves for each allele and reference gene were included on all plates consisting of serial 10-fold dilutions (six different dilutions) of pCR2.1-TOPO plasmids containing the PCR amplified fragments. The plasmids were diluted in ultrapure water containing 10 ng/µL yeast tRNA (Invitrogen) and 5 µL were added to the PCR mix generating final concentrations ranging from 2×10^−2^ −2×10^−7^ ng/µL. Amplification efficiencies were calculated by the method of Pfaffl [53] and only primer combinations with an efficiency exceeding 1.96 per cycle (actual range: 1.92–2) were accepted. For each sample, the cDNA copy number was calculated from the standard curve and divided by the copy number of the reference gene.

### Primer design and software

Databases containing all available HLA-A, -B and -C allele sequences obtained from release 2.24.0 (2009) of the IMGT/HLA database (http://www.ebi.ac.uk/imgt/hla/) were compiled to allow for the matching of input sequences to HLA subtypes [51]. An HLA-specific primer-design algorithm was conceived to facilitate the search for forward and reverse HLA allele-specific primers that would satisfy a number of requirements such as a 3′ base difference, 21-mer length, melting temperature of 66°C, amplified fragment length of 70-120 bp and no cross-reactivity with other alleles in the HLA database. HLA-ABC allele-specific sequences obtained for DD8 cells were aligned using the AlignX algorithm of VectorNTI Advance version 10 software (Invitrogen). We also designed HLA allele-specific primers using OLIGO 6.0 (Molecular Biology Insights).

### Ethical permission

The study was reviewed and approved by the Ethical Committee for the Region of Southern Denmark, with the issue no. 2003-41-3206, 2008-00-92. Written informed consent was obtained from the healthy blood and bone marrow donors joining the study with respect to sampling and establishment of MSC cell lines.

## Supporting Information

Table S1 List of Antibodies.
(0.02 MB XLS)
